# Unrecognized Subclinical Infection with Tickborne Encephalitis Virus, Japan

**DOI:** 10.3201/eid2310.170918

**Published:** 2017-10

**Authors:** Kentaro Yoshii, Reiji Kojima, Hiroshi Nishiura

**Affiliations:** Hokkaido University, Sapporo, Japan (K. Yoshii);; Japan Self-Defense Forces Sapporo Hospital, Sapporo (R. Kojima);; University of Yamanashi, Yamanashi, Japan (R. Kojima); Hokkaido University, Sapporo (H. Nishiura)

**Keywords:** Subclinical infection, tick-borne encephalitis virus, seroepidemiologic study, flaviviruses, neutralizing antibody, Japan, viruses, vector-borne infections, ticks, Japanese encephalitis virus, zoonoses

## Abstract

During early 2017, we conducted a seroepidemiologic investigation for tickborne encephalitis virus among 291 Japan Self-Defense Forces members in Hokkaido. Two (0.7%) tested positive. Neither had clinically apparent symptoms after removing ticks.

Tickborne encephalitis virus (TBEV; genus *Flavivirus*, family *Flaviviridae*) persists in ticks and wild animals, including wild rodents and shika deer (*1*,*2*). Geographically, the virus is widely spread across Eurasia and annually causes ≈10,000 clinically apparent cases in humans (*3*). In Japan, tickborne encephalitis is notifiable. Two confirmed cases, both in Hokkaido in the northernmost prefecture, have been reported. The first confirmed case was serologically diagnosed in 1993 (*4*); the second case was confirmed in 2016.

During the 20-year gap between cases, serologic and virologic surveys of wild animals (*5*,*6*) clarified that TBEV has been maintained in animal populations, especially in wild rodents in Hokkaido. Given the continued ecologic findings of virus activity in animals, it is plausible that humans have acquired TBEV infection, especially persons frequently exposed to ticks, including Japan Self-Defense Forces (JSDF) members of the Northern Army. We report the result from a pilot seroepidemiologic study of JSDF members conducted to determine the presence of unrecognized infections and to crudely measure the frequency.

We recruited 291 JSDF members who belong to the Northern Army and who received tick bites during ground activities (*7*). During their general health screening in February and March 2017, participants were asked to provide an additional 2 mL of serum for laboratory testing and to answer a questionnaire about the frequency of tick bites. We conducted neutralizing antibody testing using the virus isolated from Hokkaido in 1993 (*4*). We determined a serum sample to be TBEV positive if >50% plaque reduction compared with healthy human control serum was observed. We defined neutralizing titer as the reciprocal of the highest dilution of serum. To differentiate TBEV infection from Japanese encephalitis virus (JEV) infection, which occurs in southwestern Japan, we also conducted neutralization testing for JEV on all TBEV-positive samples.

The Medical Ethics Committees at the Graduate School of Medicine, Hokkaido University and JSDF Sapporo Hospital approved this study. The research team explained to participants that the enrollment was voluntary and gave participants the right to withdraw. We obtained written informed consent from participants, and no names were assigned to serum samples or questionnaires.

Participants ranged in age from 35 to 54 years (mean 43.3 years). A total of 288 (99.0%) of the 291 participants were men. Ninety-two (31.6%; 95% CI 26.5%–37.1%) participants appeared to have been bitten by ticks >1 time during the previous 10 years; participants were bitten a mean of 1.4 (SD ± 1.1) times. Two (0.7%; 95% CI 0.0%–1.7%) persons appeared to have been infected with TBEV; both were negative for JEV, the only other flavivirus in Japan. The TBEV-positive participants were men 42 and 48 years of age who had been bitten 3 and 1 times, respectively, within the previous 10 years (Table). Neither man complained of symptoms of TBEV infection, such as high-grade fever, headache, nausea, or paralysis after tick removal; however, the 48-year-old participant noted right knee joint pain, inguinal lymph node swelling, and low-grade fever, which he attributed to the remaining body part of a tick.

**Table:** Neutralizing antibody titers against TBEV and JEV among members of the Japan Self-Defense Forces screened in early 2017*

Patient age, y	Received tick bite in previous 10 y	Antibody titer†	
TBEV	JEV
42	3 times	80	<20
48	1 time	40	<20

*JEV, Japanese encephalitis virus; TBEV, tickborne encephalitis virus. †Neutralizing titer was defined as the reciprocal of the highest dilution of serum.

These 2 unrecognized subclinical TBEV infections were serologically diagnosed, demonstrating that humans who are particularly at risk for tick bites are partly asymptomatically infected with TBEV in Hokkaido. Because flaviviruses are known to serologically cross-react with other close flaviviruses (*8*), we tested serum against JEV, the only other endemic flavivirus in Japan, and successfully excluded its possibility. The antibody titer was lower than that in persons with clinically apparent cases (e.g., >1,600), perhaps because the virus replication was limited among subclinical cases or antibody had decayed since infection. 

Our findings echo similar cross-sectional survey results among persons recently bitten by ticks in Xinjiang and Inner Mongolia, China (*9*). Although the estimated frequency in Japan was as low as 0.7%, this figure should not be regarded as small, considering that >30,000 persons serve in the Northern Army. In addition, frequently bitten persons include not only JSDF members but also dairy farmers, foresters, and hikers. Seroepidemiologic surveys with greater sample size and broader scope of study participants are needed to identify persons at high risk for infection and determine the pros and cons of specific countermeasures, including vaccination (*10*). Such surveys also are needed to measure the virulence of TBEV of the so-called Far-Eastern subtype because the detection of subclinical or mild cases may lead to an overall decrease in its case-fatality risk, which is perceived as high (*1*).
